# Developing Gene-Based Personalised Interventions in Autism Spectrum Disorders

**DOI:** 10.3390/genes13061004

**Published:** 2022-06-02

**Authors:** Christine M. Freitag, Antonio M. Persico, Jacob A. S. Vorstman

**Affiliations:** 1 Department of Child and Adolescent Psychiatry, Psychosomatics and Psychotherapy, Autism Research and Intervention Center of Excellence, University Hospital Frankfurt, Goethe-Universität, 60528 Frankfurt am Main, Germany; 2 Department of Biomedical, Metabolic and Neural Sciences, University di Modena e Reggio Emilia, 41225 Modena, Italy; antonio.persico@unimore.it; 3 Department of Psychiatry, University of Toronto, Toronto, ON M5S 1A1, Canada; jacob.vorstman@sickkids.ca; 4 Program in Genetics and Genome Biology, Research Institute and Autism Research Unit, The Hospital for Sick Children, Toronto, ON M5G 0A4, Canada

Autism spectrum disorder (ASD) is a neurodevelopmental disorder with onset in early childhood. While highly heterogeneous, the core manifestations always include persistent difficulties in social interaction and communication, as well as a pattern of restricted interests, repetitive behaviours, and abnormal sensory processing [1]. In addition, psychiatric comorbidity is high [2], and there are genetic risk overlaps with some other mental and neurodevelopmental disorders. In the vast majority of cases, the condition persists into adulthood [3], albeit with various behavioural features and variable mental and somatic comorbidity over a given lifespan. ASD is associated with high societal, educational, and health care costs, and, in many cases, a dramatic impact on the quality of life of patients and their families. ASDs are highly heritable [4], and a multitude of genetic studies have been published. In addition, more recent reviews also emphasize the role of genetic and environmental factors in the pathophysiology of ASD [5,6], which are mediated by lasting epigenetic changes. The genetic architecture of ASD comprises common and rare variations as well as cytogenetic disturbances, such as copy number variations, translocations, inversions, and numerical chromosomal aberrations [7]. Based on the genes affected and the respective functional effects, the idea of personalised medicine is to eventually use that information for the development of targeted treatments or towards the ability to predict the response to a specific intervention, mainly pharmacological but also psychosocial, given the individual’s genetic and environmental risk constellation. The current Special Issue aims to highlight some core aspects regarding basic and applied science approaches in advancing this field of science.

Currently, psychopharmacological treatment in ASD can improve many comorbid neurodevelopmental disorders, such as attention-deficit/hyperactivity disorder or aggressive behaviour, and the core symptoms of restricted and repetitive behaviours [8,9]. No pharmacological options targeting social interaction and communication are available. Social communication and other strongly relevant targets of intervention in ASD [10], such as adaptive behaviour, cognitive and language development, or quality of life may be improved by early behavioural intervention [11]. Still, individual outcomes are highly variable, even with the same kind of psychosocial intervention approach. A better understanding of the pathophysiological mechanisms underlying this broad range of symptoms and abilities, as well as their longitudinal course, is a crucial first step towards the development of personalised treatments.

Given the heterogeneity regarding the ASD phenotype and its underlying etiology, such as diverse genetic variation and additional environmental risks with the related neurobiological mechanisms, discovering new pharmacological treatments for the condition is a huge challenge. This challenge is at the heart of this Special Issue. Here, we have collected a set of contributions providing state-of-the-art coverage, ranging from the theoretical framework, linking genetics to human behaviour and therapy, to initial practical examples of how genetics can provide valuable insights into the personalized clinical management of autistic individuals. To introduce the papers of this Special Issue, a broad summary of the many challenges related to the development of personalised medicine in ASD is given here. In the final statement from the editors, the specific contributions of the articles included in this Special Issue will be summarised.

## 1. Outcomes in Studies with Biological Models

Due to the limitations of comparable clinical outcome measures in ASD, genetic research aiming to describe underlying biological mechanisms has most often taken the gene-to-behaviour approach, as a rule, via intermediary phenotypes. In the case of cell models, morphologocial neural change, differential gene expression and other functional approaches to neural development are used [12]. These outcomes may be compared between cell models derived from individuals with or without a respective genetic risk factor (iPSC-models), or—in a more standardised and less individualised fashion—specific knockouts are compared to wild-type neural cell models. Regarding animal models, besides brain morphological, gene expression, and functional outcomes over development, social communication outcomes may also be studied, such as sociability, social novelty, or ultrasonic vocalization [13]. Still, the interpretation of these results in relation to children, youth, and adults with ASD is a major challenge, which has been discussed by recent reviews taking different approaches, such as gene-based [14] or function-based [15] approaches. These challenges and limitations must be kept in mind when interpreting cell- and animal-model-based study results. Still, biological models remain indispensable to develop a mechanistic concept for the (joined) effect of mutations or common variation on course and outcome of ASD.

## 2. Joint Mechanisms across Models

A promising approach in delineating the functional consequences of genetic risk factors aims to study molecular or neural mechanisms, which show overlapping characteristics between species. For example, the role of mutations in the mTOR or RAS signalling pathways show similar neuropathological characteristics in human and rodent models [16]. Gene expression pattern, neural synaptic function, or the imbalance of inhibitory and excitatory neural functions are other well-studied aspects which can be compared between rodent and human models [13,17,18]. Additionally, the role of somatic mutations has been the focus of recent exciting developments, especially in the field of treatment-resistant epilepsy, as documented both in humans and in animal models [19,20]. In relation to these joint mechanisms, drugs can be studied. These may first be tested in cells and subsequently in rodent models. Intermediary phenotypes (such as gene expression pattern, neural structure, or functional phenotypes) may be chosen first to study change by intervention. These outcomes are closely related to the involved mechanisms and—if an intervention is shown to be effective here—it follows that the specific drug or intervention may support the reestablishment of the typical expression pattern, neural structure, or function. If this first step has been successful, then more distal outcomes need to be chosen, which are more closely related to core ASD symptoms in the areas of social communication or stereotyped and repetitive behaviours.

## 3. Testing Compounds and Innovative Intervention Approaches

To test new pharmacological compounds related to the specific genetic background of an individual, pharmacological information has to be linked with the individual’s molecular findings [21]. The availability of known compounds with certain functions is crucial here. Several papers in the present Special Issue discuss these ideas, for example, through presenting possible new pharmacological options for carriers of specific genetic syndromes [13,22], or the authors translate neurobiological findings in relation to specific genetic syndromes into clinical care [23]. Finally, in addition to drug repurposing, gene therapies also may be developed in the future as new treatment option for ASD [24].

## 4. Translation to Clinical Trials

Currently, clinical trials may either be carried out in individuals with a specific monogenetic disorder or microduplication/-deletion, which is often accompanied by ASD, such as fragile-X syndrome (FraX). This approach has been followed by large consortia and is described in several articles of this Special Issue. A recent comprehensive review has delineated the road to precision psychiatry by translating genetics into disease mechanisms [25]. Still, from the clinical point of view, the need for personalised approaches arises with specific phenotypic features of an individual, related to ASD core or comorbid symptoms, age, and cognitive and language development. From this clinical point of view, guidance on targeted molecular diagnostic and derived pharmacological approaches directed at specific patterns of the phenotype in relation to the individuals age, IQ, and specific cognitive abilities is of importance [26]. To answer this question, the importance of large genotype–phenotype datasets, taking a lifespan perspective, and including biological, genetic, environmental risk or protective information, and clinically important outcome measures, is increasingly recognized. The rarity of single genetic disorders, coupled with the need for large sample sizes to attain sufficient statistical power, has made it imperative to collaborate across research sites and country borders, as is increasingly apparent from initiatives such as EU-AIMS and EU-AIMS2-Trials, organising the European Autism Genomes Registry (EAGER), the ASD working group of the Psychiatric Genomics Consortium (ASD-PGC), and the Genes To Mental Health (G2MH) initiative [27,28,29].

## 5. Choosing Relevant Outcomes for Clinical Trials in ASD

Recent developments in the design of clinical trials for individuals with ASD have emphasized the strong relevance of change-sensitive and patient-centred outcome measures, which should be used in clinical trials with individuals with ASD. A seminal article implementing qualitative and quantitative methods has reviewed the state of the art on outcome measures in young children with ASD [10]. Qualitative interviews showed that parents of children with ASD have slightly different expectations for the desired outcomes of interventions for their young children compared with the therapists. This finding shows that it is important to add the parents’ and patient’s perspective to clinical research in ASD. Thus, recently, a broad conceptual model on the impact of living with ASD has been developed in interaction with parents of young children, youth, and adults with ASD [30]. The model states detailed patient-centred aspects of (1) core ASD symptoms in the areas of communication, socialization, and restrictive and repetitive patterns of behaviour, including sensory issues; (2) associated physical, cognitive, emotional, and behavioural symptoms; and (3) measures on the impact of ASD on activities of daily living, schoolwork, and social life. The most frequently reported concepts were related to social communication, i.e., conversation skills, social interaction, and improved expression and processing of emotions. For the development of gene-based, personalised approaches in ASD, it is important to keep in mind that these social outcomes are, of course, highly species-specific outcomes, so cell models cannot directly be tested with regard to such outcomes. Additionally, the results of animal models can at most indirectly indicate a possible impact on social communication outcomes in ASD. In contrast, stereotyped repetitive behaviours, including sensory sensitivity, are more easily captured by animal models [31]. Thus, an approach integrating results from human studies on phenotype–genotype relationships, idealistically with a longitudinal approach, with results concerning the genotype–intermediary phenotype relationship based on cell and animal models will eventually allow a personally meaningful translation of personalised approaches into the clinic. Another option may be a secondary analysis of the highly variable outcomes of randomised controlled trials, including participants with the respective genetic information [32], followed by functional studies to elicit possibly related molecular pathways.

## 6. Conclusions

Personalised medicine, especially pharmacotherapeutic approaches, based on individual genetic burden, has made steady progress over recent years. The combination of phenotypically and genetically informed personalised approaches, and in relation to pharmacological and psychosocial intervention, is another area of importance for the clinicians, the affected individuals, and their families.
